# Supplementary material for: Drug- and Cell-Type-Specific Effects of ROCK Inhibitors as a Potential Cause of Reticular Corneal Epithelial Edema
Source: Cells. 2025 Feb 11;14(4):258. doi: 10.3390/cells14040258 (PMC11853206; doi:10.3390/cells14040258)
Supplement: Supplementary file 1 [file cells-14-00258-s001.zip › cells-3440777-supplementary.pdf]

Supplementary Table S1. SYBR green primers used for qRT-PCR; T_{an}, annealing temperature.

Gene symbol	Accession	Product length	T_{an}	$MgCl_2$ (mM)	Sequence 5' - 3'
AQP1	NM_198098.4	142	61	3.0	CCATCCTCTCAGGCATCACCTC<br>GTAGTAGCCAGCACGCATAGCA
AQP3	NM_004925.5	159	62	3.0	GAGATGCTCCACATCCGCTACC<br>AAAGCCAAAGGCCAGGTTGATG
AQP5	NM_001651.4	169	62	3.0	CTGCTCCGGGCTTTCTTCTACG<br>AGGTCAGAATCAGCTCCACCAC
AQP11	NM_173039.3	221	61	3.0	CCAAGCTTCGTATCCACCTGCT<br>ATGCAGCCATGGAAGGAAAAAGC
ATP1A1	NM_000701.8	179	61	3.0	TGCTTTGTTGGGCTCATCTCCA<br>TCTTCCACGGTCTCATTGCCTT
ATP1A4	NM_144699.4	238	61	3.0	ACTTTTCCAGCAGGGCATGAGA<br>GTACGTCTCCCTTTCCACCCAG
ATP1B1	NM_001677.4	115	61	3.0	GAAGAAGGAGTTTCTGGGCAGG<br>CAGCATCACTTGGATGGTTCCG
ATP1B3	NM_001679.4	116	61	3.0	AGCTCTTCATCTACAACCCGACC<br>TGAGAAGAGTGCAGCCAGGAAC
ATP2A1	NM_004320.6	221	61	3.0	ACCGCTGTAACTATGTGCGAGT<br>TACACCCACGAATGTCAGGTCC
ATP2B1	NM_001682.3	237	61	3.0	GATCACGCTGAAAGGGAGTTGC<br>AGGAGCATCATCTTCGGCATCA
CA2	NM_000067.3	157	61	3.0	CTGATGGACTGGCCGTTCTAGG<br>TCCAAGGATTCAGGAAGGAGGC
CDH1	NM_001317184.2	196	62	3.0	CAGGATGGCTGAAGGTGACAGA<br>AGAGCACCTTCCATGACAGACC
CDH2	NM_001792.5	161	60	3.0	ACAGAATCGTGTCTCAGGCTCC<br>TGTGGGATTGCCTTCCATGTCT
CLDN1	NM_021101.5	189	61	3.0	GTGCTTGGAAGACGATGAGGTG<br>CAGCCAGTGAAGAGAGCCTGAC
CLDN2	NM_020384.4	115	62	3.0	TTGTACTTCGCTCCCCTCCCTC<br>ACAAGCAGCCTCAAGAAGGCAT
DSC2	NM_004949.5	94	61	3.0	CGTCCTGTAGATCGTGAGCAGT<br>TTAGGGGCAGTGGAAGTTCTGG
DSG1	NM_001942.4	147	61	3.0	TCTTCTGAACCCGGAAACGGAG<br>AGGAGCACCTCCACAATCACAA
GAPDH	NM_002046.6	194	64	3.0	AAGGTCGGAGTCAACGGATTTGG<br>ATGACAAGCTTCCCGTTCTCAGC
GJA1	NM_000165.5	174	61	3.0	CTGAGTGCCTGAACTTGCCTTT<br>CCTTCCCTCCAGCAGTTGAGTA
HPRT1	NM_000194.3	229	58	3.5	TGCTGACCTGCTGGATTACA<br>CCTGACCAAGGAAAGCAAAG
ITGA6	NM_000210.4	111	62	3.0	TGTTGCTGTTGGTTCCCTCTCA<br>TTCTGGCGGAGGTCAATTCTGT
ITGB1	NM_002211.4	180	62	3.0	TTGTAGCTGGTGTGGTTGCTGG<br>TGACCACAGTTGTTACGGCACTC
ITGB4	NM_000213.5	185	62	3.0	CCACGACTCTCGCCTGACTG<br>GGTTGGGGATGTTGAGCCGATG

KRT3	NM_057088.3	186	60	3.0	CGGGAACAGATCAAGACCCTCAA
					GCTCCGCAGGTAGTTGATGTGA
KRT12	NM_000223.4	171	60	3.0	CTGGAGATTGAGACCTACCGCC
					ACCATTCACCATCTCCTGCACA
OCLN	NM_002538.4	111	61	3.0	GTGATGAGCTGGAGGAGGACTG<br>AGCTCTTGTATTCCTGTAGGCCA
PLEC	NM_201384.3	175	61	3.0	CAAGTGGGTCAACAAGCACCTC<br>CAGGGCAATCTGGACATTCTGC
RPLP0	NM_001002.4	235	61	3.0	GCAGCATCTACAACCCTGAA<br>GCAGATGGATCAGCCAAGAA
SLC4A4	NM_003759.4	70	61	3.0	ATGGAGTGGAAGGAAACAGCCA<br>TCCATCTTTCCCCACCCTGTTC
SLC4A7	NM_003615.5	203	61	3.0	AACAGATGCAAGCAGCCTTGTG<br>CATTGCTGGGGTTTGGAGGTTC
SLC4A11	NM_032034.4	75	61	3.0	ATTCCTGTGAGGTTCGGTTCGT<br>CCTCCATCGCAGTCTTAGTGCT
SLC7A14	NM_020949.3	173	61	3.0	GGACCTCATCTCTCTTGGCGTT<br>TGGGGACTCGAACTCCAAACTC
SLC9A1	NM_003047.5	235	61	3.0	AGAACGCTCGATTGGGGATGT<br>CTCCGGGACGATGCTTGAGAT
SLC12A1	NM_000338.3	107	61	3.0	TTCAGGGATGGTGTCCGAAGTC<br>GTCAAGGGAGCTTTCCTCCAGT
SLC12A2	NM_001046.3	137	61	3.0	TCTGGTGGCTTTTTGATGATGGAG<br>CGCTCTCCGGTCATGGTCTATTC
SLC16A1	NM_003051.4	240	61	3.0	GGAGCTTTCATTTCCATCGGCT<br>AGAAGCTGCAATCAAGCCACAG
SLC16A3	NM_004207.4	211	61	3.0	CTGGATCTCCTCCATCCTGCTG<br>CTGGAAGTTGAGTGCCAAACCC
SLC16A7	NM_004731.5	200	61	3.0	CCCCAAAGCTGTCACCGTATTC<br>CCAACACCATTCCAAGACAGCA
SLC17A5	NM_012434.5	126	61	3.0	ACCCTGTTCACTCCCATTGCTG<br>GGGAGCCCAAGAAGACCACAT
TJP1	NM_003257.5	191	62	3.0	TCCTTTTCCTGCTTGACCTCCC<br>ATCCACAACACGGAACACCTCT
TJP2	NM_004817.4	197	62	3.0	GTGTTGAGAGACAGCCAGCAGA<br>GGCGTGTCCTCTCTGGAACTTG
TP63	NM_003722.5	95	61	3.0	TGCAGCATTGTCAGTTTCTTAGC<br>TGCTCAATCTGATAGATGGTGGT

Supplementary Table S2. List of antibodies used

**Antibody (clone), Host species**	**Antibody concentration**	**Antibody source**
Connexin 43, rabbit	1:100	Cell Signaling
Cytokeratin pan, rabbit	1:100	Abcam
Cytokeratin 3/76 (AE5), mouse	1:50	Millipore
Cytokeratin 12 (EPR17882), rabbit	1:30	Abcam
Cadherin-1 (36), mouse	1:100	BD Transduction
Cadherin-2, rabbit	1:100	Abcam
Integrin α6 (GoH3), rat	1:100	Millipore
Na^+/K^+-ATPase (D4Y7E), rabbit	1:100	Cell Signaling
Occludin (E6B4R), rabbit	1:150	Cell Signaling
P63 alpha, rabbit	1:100	Cell Signaling
Phalloidin-Alexa Fluor 555	1:20	Cell Signaling
Vimentin (EPR3776), rabbit	1:1000	Abcam
ZO-1 (1/ZO-1), mouse	1:25	BD Biosciences

Supplementary Figure S1. Effect of ripasudil and netarsudil on corneal endothelial cell migration

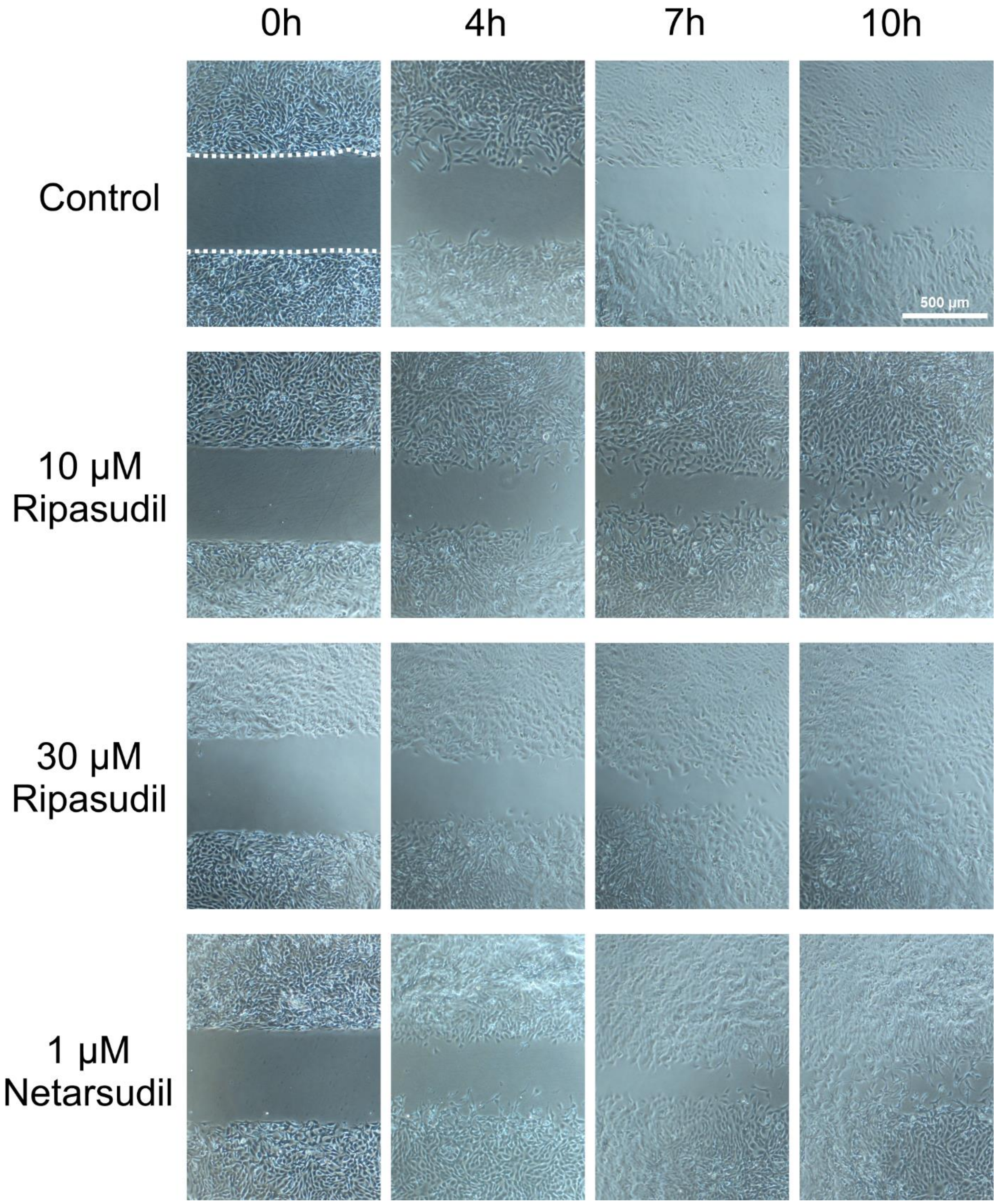

Effect of ripasudil and netarsudil on corneal endothelial cell migration (phase contrast images; magnification x100).

Supplementary Figure S2. Effects of ripasudil and netarsudil on corneal endothelial pump function.

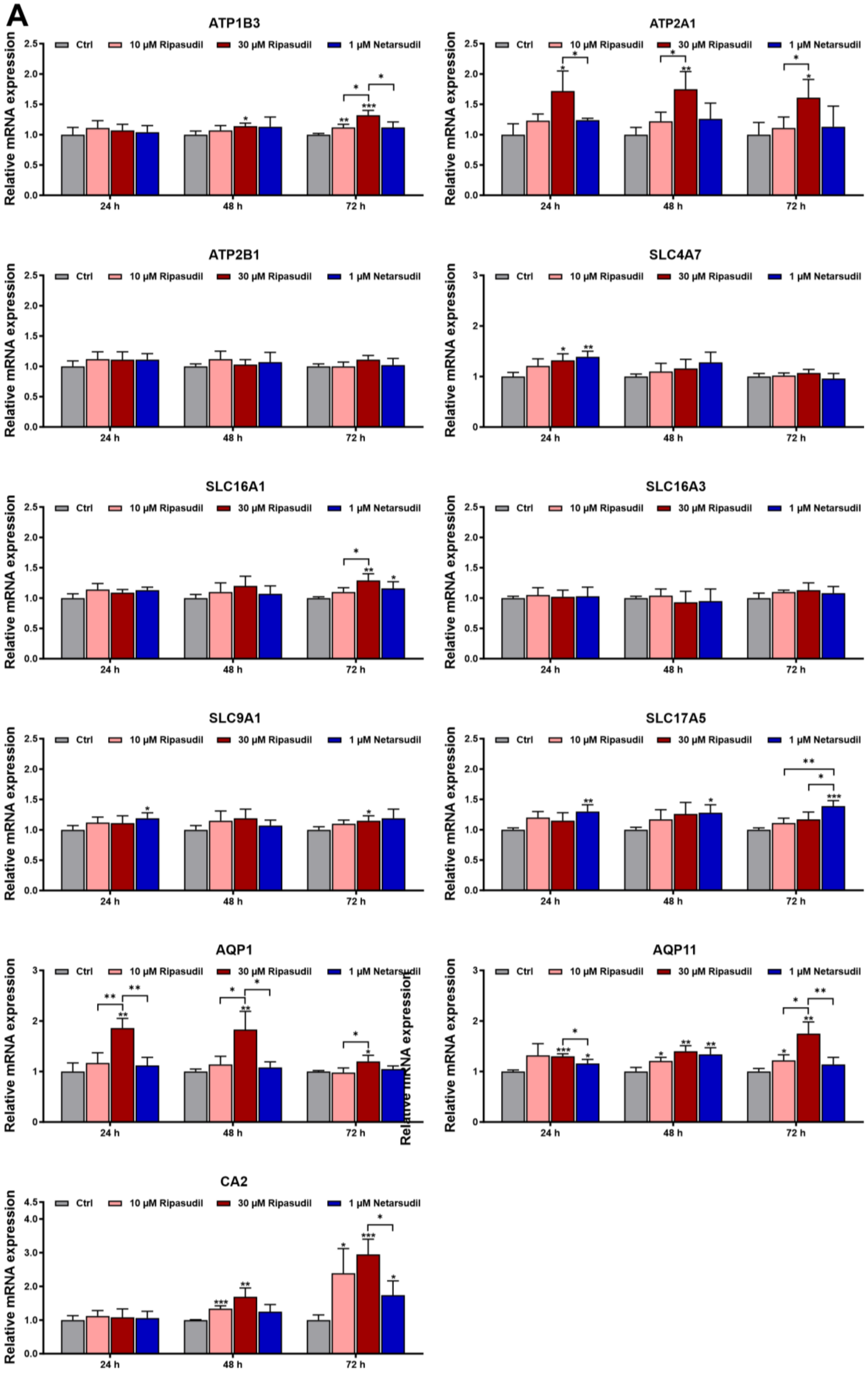

Quantitative real-time PCR analysis of primary human corneal endothelial cells treated without or with ripasudil (10 and 30 µM) and netarsudil (1 µM) for 24 to 72 hours, respectively (n=6) showing relative mRNA expression levels of ATP1B3 (Na,K-ATPase beta-3 subunit), ATP2A1 (ATPase Sarcoplasmic/Endoplasmic Reticulum Ca2+ Transporting 1), ATP2B1 (ATPase Plasma Membrane Ca2+ Transporting 1), SLC4A7 (solute carrier familiy 4 member 7), SLC16A1 and SLC16A3 (solute carrier familiy 16 member 1 and 3), SLC9A1 (solute carrier familiy 9 member 1), SLC17A5 (solute carrier familiy 17 member 5), AQP1 and AQP11 (aquaporin 1 and 11), and CA2 (carbonic anhydrase 2). Data are normalized to GAPDH (glyceraldehyde-3-phosphate dehydrogenase) and HPRT1 (hypoxanthine phosphoribosyltransferase 1) and expressed as means ± SD relative to controls set to 1 (*p<0.05, **p<0.01,***p<0.001; unpaired t-test).

Supplementary Figure S3. Characterization of primary human corneal epithelial cells

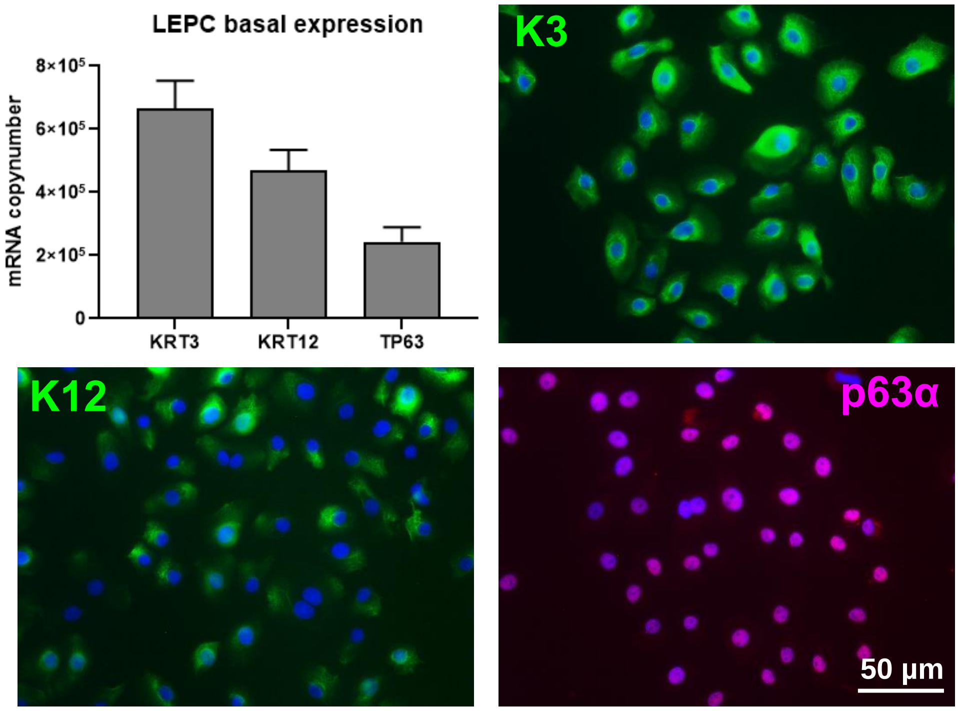

Quantitative real-time PCR analysis of cultured primary human corneal epithelial cells (n=3) showing absolute mRNA expression levels of KRT3 (Keratin 3), KRT12 (Keratin 12) and TP63 (Tumor Protein P63). Immunocytochemical analysis shows cytoplasmic expression of keratin 3 (K3) and 12 (K12) as well as nuclear expression of p63 alpha on the protein level (DAPI nuclear counterstain in blue).
